# Alterations in local activity and functional connectivity in patients with postherpetic neuralgia after short-term spinal cord stimulation

**DOI:** 10.3389/fnmol.2022.938280

**Published:** 2022-08-11

**Authors:** Xiaochong Fan, Huan Ren, Chunxiao Bu, Zhongyuan Lu, Yarui Wei, Fuxing Xu, Lijun Fu, Letian Ma, Cunlong Kong, Tao Wang, Yong Zhang, Qingying Liu, Wenqi Huang, Huilian Bu, Jingjing Yuan

**Affiliations:** ^1^Department of Pain Medicine, The First Affiliated Hospital of Zhengzhou University, Zhengzhou, China; ^2^Department of Magnetic Resonance Imaging, The First Affiliated Hospital of Zhengzhou University, Zhengzhou, China; ^3^Department of Anesthesiology, The First Affiliated Hospital Sun Yat-sen University, Guangzhou, China; ^4^Department of Anesthesiology, Pain and Perioperative Medicine, The First Affiliated Hospital of Zhengzhou University, Zhengzhou, China

**Keywords:** spinal cord stimulation, pain, postherpetic neuralgia, functional magnetic resonance imaging, mechanisms of action, emotion

## Abstract

**Introduction:**

The efficacy of short-term spinal cord stimulation (stSCS) as a treatment for neuropathic pain in patients with postherpetic neuralgia (PHN) has already been validated. However, the potential alterations in brain functionality that are induced by such treatment have yet to be completely elucidated.

**Methods:**

This study use resting-state functional magnetic resonance imaging (rs-fMRI) to detect the changes in regional homogeneity (ReHo) and degree centrality (DC) related to stimulator-induced pain relief in patients with PHN. A total of 10 patients with PHN underwent an MRI protocol at baseline and after stSCS. Alterations in ReHo and DC were then compared between baseline and after stSCS. We investigated the relationship between clinical parameters and functional changes in the brain.

**Results:**

Clinical parameters on pain, emotion, and sleep quality were correlated with ReHo and DC. ReHo and DC were significantly altered in the middle temporal gyrus, precuneus, superior frontal gyrus, supramarginal gyrus, inferior parietal lobule, rolandic operculum, middle occipital gyrus, superior parietal gyrus, and the precentral gyrus after stSCS. A significant correlation was detected between ReHo changes in the middle occipital gyrus, precuneus, inferior parietal gyrus, and changes in pain, emotion, and sleep quality. A significant negative correlation was detected between DC changes in the middle temporal gyrus, rolandic operculum, supramarginal gyrus, precuneus, inferior parietal gyrus, and changes in pain, emotion, and sleep quality.

**Conclusion:**

This study found that stSCS is able to induce ReHo and DC changes in patients with PHN, thus suggesting that stSCS can change brain function to alleviate pain, sleep, and emotional disorder.

## Introduction

Herpes zoster (HZ) results from the reactivation of varicella-zoster virus and manifests as a vesicular, painful, dermatomal rash (Johnson and Rice, 2014). The annual incidence of HZ is 3.4 cases per 1,000 persons (Insinga et al., 2005). Postherpetic neuralgia (PHN) is the most frequent complication of HZ. After the age of 50 years, 20% of patients with HZ will develop PHN (Brisson et al., 2001). PHN results in lasting physical discomfort, mental suffering. PHN reduces the quality of life and increases the financial burden.

Pain is a multidimensional experience that covers diverse and integrated brain functions. Local brain chemistry, construction, and function reorganized in patients with neuropathic pain (Li et al., 2020). Due to the complicated pathogenesis of PHN, we incompletely understand about how this condition affects the function of the brain. A study has revealed abnormal local brain activity in patients with PHN (Dai et al., 2020).

The treatment is based on the control of symptoms (Johnson and Rice, 2014). In addition to medicinal therapy (Moore et al., 2014), other forms of interventional therapies have been shown to effectively alleviate pain, such as nerve block (Kuo et al., 2016), pulsed radiofrequency (PRF) (Han et al., 2020), and spinal cord stimulation (SCS). The oral medications have systemic and cognitive adverse effects. Although nerve block is effective, its duration is transient (Kuo et al., 2016). The short-term SCS (stSCS) which based on the electric stimulation, is an effective clinical treatment. The stSCS is more effective than PRF for patients with PHN (Wan and Song, 2021).

Since the day that SCS was introduced, researchers have been investigating the mechanism of action. Supraspinal structures may play a role in the pain alleviating effects of SCS (Meyerson and Linderoth, 2006). However, potential functional brain alterations associated with the application of SCS as a treatment for PHN remain unclear. Functional magnetic resonance imaging (fMRI) is a powerful non-invasive modality that can map the areas of the brain involved in pain perception and modulation (Tracey, 2011). Spontaneous local and long-range brain activity can be quantitatively measured by regional homogeneity (ReHo) (Zuo et al., 2013) and degree centrality (DC), respectively.

The first proposed that ReHo could be a methodology to characterize functional homogeneity of resting-state fMRI (rs-fMRI) signals by Zang et al. (2004). ReHo reflects the local coherence of local spontaneous neuronal activity. Degree centrality (DC) shows the number of direct connections with the rest of brain (Wu et al., 2020). A brain regional node with high centrality is potentially a hub for information transition. Previous research has not considered the alteration in ReHo during SCS treatment. With regard to functional connectivity, the study reported that connection strength changed in patients treated with SCS for neuropathic pain (De Groote et al., 2020). Based on the available information, we speculate that ReHo and DC may be altered when stSCS is utilized to treat PHN. Additionally, we explore the potential key mechanism for rational use of stSCS to treat PHN.

To address this gap of knowledge about the mechanisms responsible for the action of stSCS therapy on patients with PHN, we studied the alterations in ReHo and DC by rs-fMRI in patients with PHN who were treated with stSCS. Then, we investigated whether there is an association between clinical data and functional brain mapping changes in patients with PHN who were treated with stSCS.

## Materials and methods

### Participants

We recruited nineteen right-handed patients diagnosed with PHN, whose pain cannot be alleviated only by conventional medications, so they receive the therapeutic procedure of stSCS, from the First Affiliated Hospital of Zhengzhou University between January 2021 and October 2021. All participants provided written informed consent prior to participating in this study. A total of nine patients withdrew their consent; therefore, our final analysis featured 10 patients.

This study was approved by the Medical Ethics Committee of the First Affiliated Hospital of Zhengzhou University, Zhengzhou, China (reference no.: 2020-KY-0299-001). The trial registration number is ChiCTR2000040239 (http://www.chictr.org.cn/listbycreater.aspx). The date of trial is November 2020.

### Study protocol

**Figure 1:**
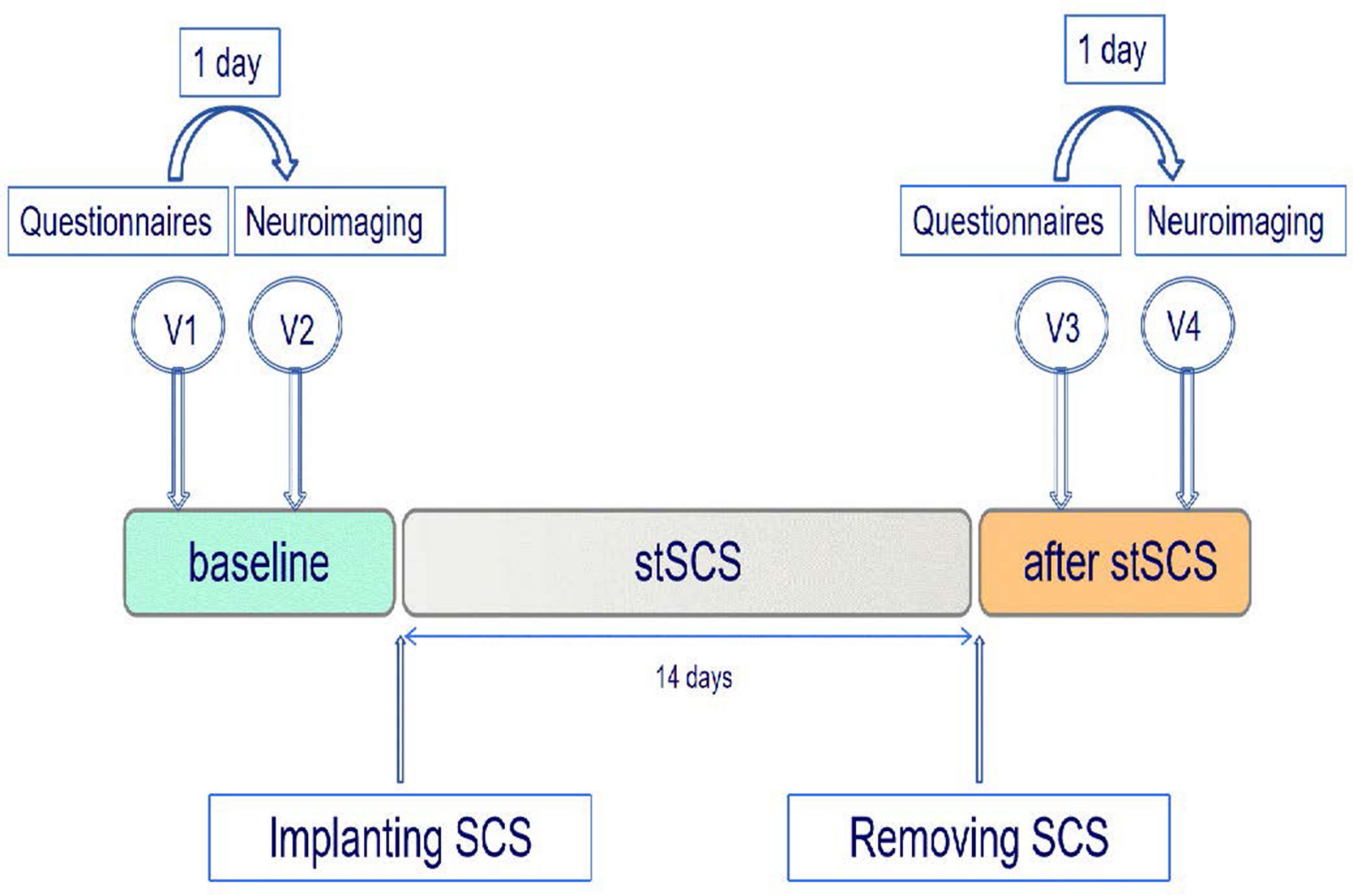
Study protocol. Patients with PHN were enrolled prior to the implantation of the SCS and were followed up after the removal of the stSCS. Each patient underwent a neuroimaging protocol prior to SCS and 14 days after SCS. SCS, spinal cord stimulation; stSCS, short-time spinal cord stimulation; V, visit.

In this exploratory and prospective cohort study, patients were included before stSCS implantation and followed up for 14 days after receiving SCS. During this period, four additional individual appointments took place (Figure 1). The first (V1) and third (V3) visits were short appointments of 30 min and were scheduled 1 day before the neuroimaging visits (V2, V4). All patients were asked to complete pain numerical rating scale (NRS) scores, the short-form McGill pain questionnaire version-2 (SF-MPQ-2), the Pittsburgh sleep quality index (PSQI), and the hospital anxiety and depression scale (HADS), during visits V1 and V3. The SF-MPQ-2 contains four subscale scores (continuous pain, intermittent pain, predominantly neuropathic pain, and affective descriptors) that were all recorded. All patients underwent a neuroimaging fMRI-protocol, during V2 (baseline) and V4 (14 days after SCS). We lost the questionnaires from one patient. Between the second and third visits, patients underwent stSCS implantation. All patients received SCS at the cervicothoracic level for PHN. The test electrode (Model 3873, US) was connected to an extension multi-lead cable (Model 355531, Medtronic, US). To program the implantable electrode, an external cable was inserted into the neurostimulator (Model 37022, Medtronic, US). The stimulation parameters were as follows: pulse width was 210–480 μs, frequency was 30–60 Hz, voltage ranged from 0 V to 10.5 V. The electrode was implanted for 14 days. Spinal electrical stimulation results in efficacy about 2 weeks. Moreover, the time of SCS more than 2 weeks will increase the risk of infection. The stimulation parameters were adjusted one time a day to ensure effectiveness. The patient had the liberty to change the amplitude with steps of 0.1 mV to provide adequate pain relief.

### MRI data acquisition

All MRI images were obtained by a 3.0T scanner (MAGNETOM PRISMA, SIEMENS, Germany) at the First Affiliated Hospital of Zhengzhou University. All subjects were requested to keep their eyes closed, and foam padding and earplugs were used to control the head movements of the participants and reduce scanning noise. At the end of scanning, patients were asked if they had fallen asleep during scanning; all patients reported that they had been awake. Resting-state functional images were collected using an echo-planar imaging sequence (repetition time = 1,000 ms, echo time = 30 ms, flip angle = 70°). A total of fifty-two transverse slices (field of view = 220 mm^2^ × 220 mm^2^, slice thickness = 2.2 mm) that aligned along the AC-PC line were acquired with a total scan time of 412 s.

### Imaging processing

The rs-fMRI data were preprocessed by the Data Processing Assistant for rs-fMRI Analysis Toolkit (DPARSF, http://rest.restfmri.net/forum/DPARSF) (Song et al., 2011) and by SPM8 software (Wellcome Department, University College of London, UK) based on MATLAB R2012a (MathWorks, USA). DPARSF was also used for the following steps: the first 10 volumes were deleted and then underwent slice-timing, realignment, spatial normalization to the Montreal Neurological Institute (MNI) space, and resampling with a 3 mm^3^ × 3 mm^3^ × 3 mm^3^ resolution. Participants with a head motion >2.5 mm of translation or >2.5° of rotation in any direction were excluded from further processing. The linear trend of the fMRI data was also removed. Band-pass filtering (0.01–0.08 Hz) was conducted to discard high-frequency physiological noise and a frequency drift lower than 0.01 Hz (Greicius et al., 2003).

Preprocessed ReHo data were analyzed as previously described (Cao et al., 2017a). First, an individual ReHo map was generated by calculating the Kendall's coefficient of concordance (KCC) of the time series of a given voxel with those of its neighbors (26 voxels) in a voxel-wise way (Li et al., 2012). Afterward, the individual ReHo maps were divided by their own global mean KCC within the whole-brain mask. Then, spatial smoothing was performed on the standardized individual ReHo map with a Gaussian kernel of 4 mm full width at half maximum (FWHM). Preprocessed data were used for DC calculations; Pearson's correlation analysis of time series was executed between each voxel and every other voxel in the entire brain. Correlation coefficients where *r* > 0.25 were summed for each voxel; then, the binary DC was obtained for each gray matter voxel (Zuo and Xing, 2014). Subsequently, spatial smoothing was performed with a 4-mm FWHM Gaussian kernel. The weighted DC of each voxel was further divided by the global mean DC for standardization. Z scores were then calculated in a voxel-wise way by subtracting the mean ReHo or DC values from each voxel's value; this was then divided by the standard deviation of the ReHo or DC values, respectively. In this way, the Z score represents a voxel's ReHo or DC value in relation to all voxels in the whole brain. Therefore, a positive Z score represents higher synchronicity (ReHo) or activity (DC) in a particular individual's brain. Similarly, a negative Z score represents lower synchronicity or activity.

### Statistical analysis

Statistical analyses were performed using SPSS software (version 21.0). Wilcoxon signed rank tests were calculated to analyze the differences in clinical outcomes between the two visits. The significance level was set at 0.05. Paired *t*-tests were conducted with SPM8 in a whole-brain voxel-wise manner for ReHo and DC comparisons between two time points. All of the results were corrected by Gaussian random field (GRF). The significance levels of the voxel and cluster were *p* < 0.005 and *p* < 0.05, respectively. Correlations between the relative change in score on the questionnaires and the relative significant change in ReHo and DC values were calculated with non-parametric Spearman correlation tests. Relative differences between baseline and treatment were calculated as (baseline-treatment)/baseline. Only clinical outcomes with a significant effect between both visits were used for correlation analysis. Multiple comparisons were corrected for correlation analysis using the Bonferroni method (*p* < 0.05/120 = 0.0004).

## Results

### Patient's characteristics

A total of 10 patients with PHN (five women and five men), and a median age of 70 years (Q1–Q3: 61.5–80) were included in this study (Table 1). Then, one patient has diabetes mellitus and five patients suffer from hypertension.

**Table 1:** Individual patient's characteristics for all patients (*N* = 10).

**Patient**	**Sex**	**Age**	**Pain**	**Pain**	**Time of**
		**(y)**	**side**	**duration**	**SCS**
				**(month)**	**(day)**
1	M	39	Right	6	14
2	M	77	Left	1	14
3	F	79	Left	3	14
4	F	62	Left	6	14
5	M	60	Right	7	14
6	F	70	Right	2	14
7	F	68	Left	4	14
8	F	87	Right	1.5	14
9	M	70	Left	1	14
10	M	83	Left	1	14

F, female; M, male; SCS, spinal cord stimulation.

### Clinical results

**Figure 2:**
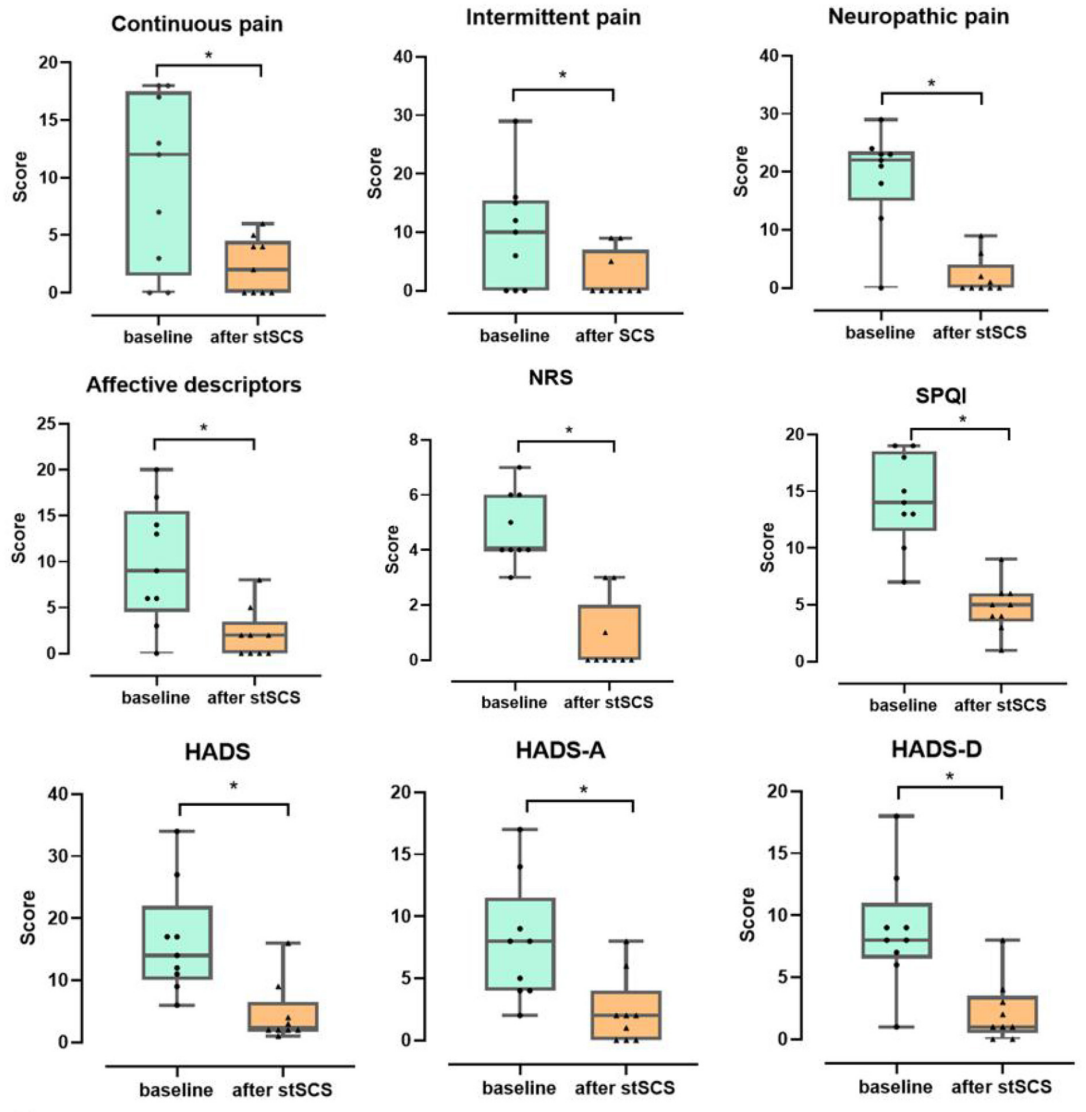
Boxplots for the clinical outcomes for all patients at baseline (in green) and after stSCS (in orange). The beginning four boxplots represent the four subscale scores of the SF-MPQ-2 (continuous pain, intermittent pain, predominantly neuropathic pain, and affective descriptors). The last two boxplots represent the two subscale scores for the HADS (HADS-A and HADS-D). NRS, numeric rating scale; PSQI, Pittsburgh sleep quality index; SCS, spinal cord stimulation. *p < 0.05.

There was a significant decrease in the total scores for the NRS score (Z = −2.694, *p* = 0.007), PSQI (Z = −2.675, *p* = 0.007), SF-MPQ-2(Z = −2.668, *p* = 0.008), and HADS (Z = −2.668, *p* = 0.008) when compared between the baseline and after stSCS treatment (Figure 2).

#### SF-MPQ-2

When considering the continuous pain, intermittent pain, neuropathic pain, and affective descriptors for SF-MPQ-2, we identified a significant decrease in scores between baseline and after stSCS: (Z = −2.371, *p* = 0.018), (Z = −2.207, *p* = 0.027), (Z = −2.527, *p* = 0.012), and (Z = −2.524, *p* = 0.012), respectively. When comparing the baseline with the status after stSCS, the median scores for the four dimensions of the SF-MPQ-2 were 12 (Q1–Q3: 1.5–17.5) to 2 (Q1–Q3:0–4.5), 10 (Q1–Q3: 0–15.5) to 0 (Q1–Q3:0–7), 22 (Q1–Q3: 15–23.5) to 0 (Q1–Q3:0–4), and 9 (Q1–Q3: 4.5–15.5) to 2 (Q1–Q3: 0–4), as shown in Figure 2.

#### Sleep quality

A significant reduction in PSQI score was identified between baseline and after stSCS (Z = −2.675, *p* = 0.007). At baseline, the median PSQI score was 14 (Q1–Q3: 11.5–18.5) and after stSCS was 5 (Q1–Q3:3.5–6), as shown in Figure 2.

#### HADS

A significant decrease in HADS score was identified between baseline and after stSCS (Z = −2.668, *p* = 0.008). At baseline, the median HADS score was 14 (Q1–Q3:10–22) and after stSCS was 2 (Q1–Q3:2–6.5). For the anxiety and depression dimensions of the HADS, we identified a significant reduction in HADS-A and HADS-D scores between baseline and after stSCS: (Z = −2.677, *p* = 0.007) and (Z = −2.673, *p* = 0.008), respectively. When comparing the baseline with after stSCS, the median scores for the two dimensions of HADS were 8 (Q1–Q3: 4–11.5) to 2 (Q1–Q3: 0–4) and 8 (Q1–Q3: 6.5–11) to 1 (Q1–Q3: 0.5–3.5), as shown in Figure 2.

#### NRS

A significant reduction in NRS score was identified between baseline and after stSCS (Z = −2.694, *p* = 0.007). At baseline, the median NRS score for PHN was 4 (Q1–Q3: 4–6) and after stSCS was 0 (Q1–Q3:0–2), as shown in Figure 2.

### fMRI results

#### Comparison of ReHo between baseline and after stSCS

**Figure 3:**
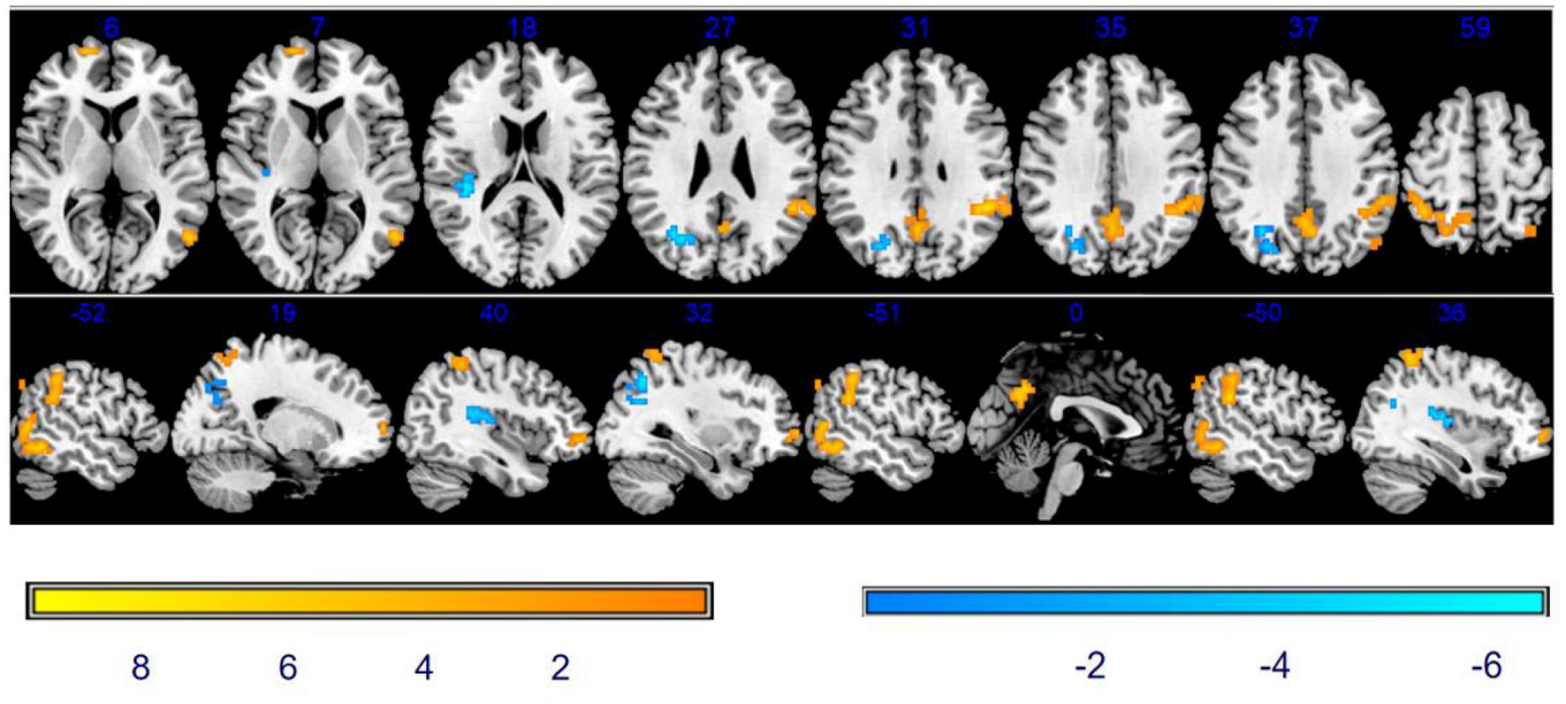
Significant differences in ReHo between baseline and after stSCS in axial and sagittal slices. The warm colors indicate a higher ReHo value whereas the cooler colors indicate a lower ReHo value at baseline and after stSCS (*p* < 0.05). Brain images are displayed in radiology convention (the left of the figure represents the right side of the patient's brain and *vice versa*).

As shown in Table 2 and Figure 3, compared with baseline, there was a significantly increased ReHo in the vast region of the left middle temporal gyrus, right superior frontal gyrus (SFG), precuneus, left supramarginal gyrus, left inferior parietal lobule (IPL), and right superior parietal gyrus. We also observed the decreased ReHo in the right rolandic operculum and the right middle occipital gyrus after stSCS treatment.

**Table 2:** Clusters of different ReHo values between baseline and after stSCS.

**Regions**	**Peak MNI**			**Peak**	**Cluster**
**Regions**	**coordinate**			***T*-value**	**size (voxels)**
	**x**	**y**	**z**		
Baseline < after stSCS
Temporal_Mid_L	−54	−63	9	8.184	61
Frontal_Sup_R	18	63	6	7.2821	39
Precuneus	−3	−60	39	8.7368	49
SupraMarginal-L	−48	−45	33	9.8058	81
Parietal_Inf_L	−36	−60	48	7.6008	46
Parietal_Sup_R	24	−51	60	6.6291	81
Baseline > after stSCS
Rolandic_Oper_R	39	−30	18	−6.5298	45
Occipital_Mid_R	27	−72	30	−6.5043	60

ReHo, regional homogeneity.

#### Correlation between ReHo and clinical results

As mentioned earlier, the NRS, PSQI scores, SF-MPQ-2, and HADS were statistically significant after stSCS therapy and were then correlated with ReHo alterations. A significant positive correlation was found between ReHo changes in the right middle occipital gyrus and changes in intermittent pain (*r* = 0.725, *p* = 0.027). A significant negative correlation was identified between ReHo changes in the left precuneus and changes in intermittent pain (*r* = −0.673, *p* = 0.047) and PSQI (*r* = −0.736, *p* = 0.024) and left inferior parietal and affective descriptors (*r* = −0.746, *p* = 0.021) and HADS-A (*r* = −0.782, *p* = 0.013). However, this level of significance was abolished after Bonferroni correction (*p* < 0.05/120 = 0.0004).

#### Comparison of DC between baseline and after stSCS

**Figure 4:**
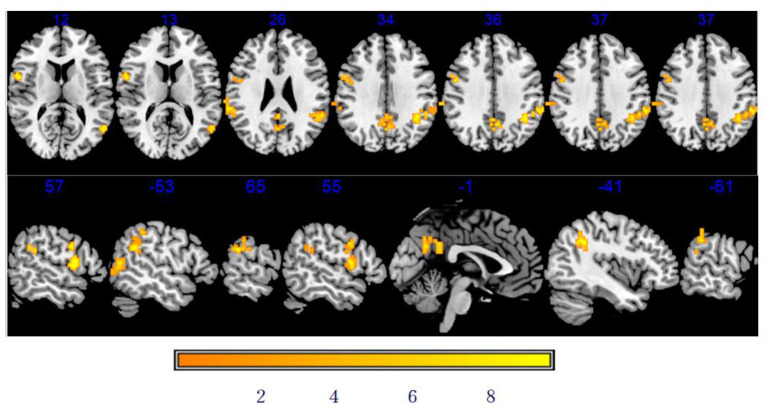
Significant differences in DC between baseline and after stSCS in axial and sagittal slices. The warm colors indicate higher DC values at baseline and after stSCS (*p* < 0.05). Brain images are presented in radiology convention (the left of the figure represents the right side of the patient's brain and *vice versa*).

As shown in Table 3 and Figure 4, there was a significantly increased DC in the vast region of the left middle temporal gyrus, right rolandic operculum, left supramarginal gyrus, right supramarginal gyrus, right precentral gyrus (PCG), left precuneus, and left inferior parietal after stSCS.

**Table 3:** Clusters of different DC values between baseline and after stSCS.

**Regions**	**Peak MNI**			**Peak**	**Cluster**
	**coordinate**			***T*-value**	**size (voxels)**
	**x**	**y**	**z**		
Baseline < after stSCS
Temporal_Mid_L	−54	−63	12	13.5598	22
Rolandic_Oper_R	54	3	12	6.5985	20
Supramarginal_L	−42	−45	30	9.6256	62
Supramarginal_R	69	−72	24	5.8351	24
Precentral_R	57	3	27	6.238	17
Precuneus_L	0	−63	27	7.2906	42
Parietal_Inf_L	−60	−42	39	7.2755	21

DC, degree centrality.

#### Correlation between DC and clinical results

The NRS, PSQI scores, SF-MPQ-2, and HADS were statistically significant after stSCS and were then correlated with DC alterations. A significant negative correlation was identified between DC changes in the left middle temporal gyrus (*r* = −0.690, *p* = 0.040), right rolandic operculum (*r* = −0.742, *p* = 0.022), right supramarginal gyrus (*r* = −0.845, *p* = 0.004) and changes in intermittent pain, left precuneus and PSQI (*r* = −0.778, *p* = 0.014), right rolandic operculum and HADS (*r* = −0.753, *p* = 0.019) and HADS-D (*r* = −0.678, *p* = 0.045), left inferior parietal (*r* = −0.686, *p* =0.041) and HADS-D. However, this level of significance did not remain after Bonferroni correction (*p* < 0.05/120 = 0.0004).

## Discussion

In our study, we investigated the changes in ReHo and DC values related to stimulator-induced pain relief in patients with PHN. ReHo and DC analysis validated the improvement in symptoms to some extent at the level of regional brain function. We also observed correlation between the change in brain function and clinical questionnaires. Our results indicate that stSCS therapy can change local activity and functional connectivity to alleviate pain, sleep, and emotional disorder.

In this study, we identified several functional brain mapping alterations related to pain following the treatment of PHN with stSCS therapy. Patients with PHN show significantly reduced ReHo levels in the temporal lobe (Cao et al., 2017b). We observed increased DC and ReHo values in the middle temporal gyrus after stSCS. The middle temporal gyrus is associated with language and semantic memory processing, visual perception, multimodal sensory integration, and pain inhibition (Kucyi et al., 2013). We also identified a significant correlation between DC and ReHo changes in the middle temporal gyrus, the right middle occipital gyrus and changes in intermittent pain. The middle occipital gyrus is related to pain and may take part in the descending pain-inhibitory mechanisms (Reis et al., 2010). The occipital lobe has been shown to be associated with sensitivity to fearful stimuli, emotion processing, levels of trait anxiety, and the sensory processing (Collignon et al., 2011). It is possible that stSCS treatment may act on these regions of brain to alleviate pain.

We found that the precuneus was related to pain intensity and sleep quality. We observed increased DC and ReHo values in the left precuneus after stSCS when compared to baseline. There was also a significant correlation between ReHo and DC changes in the left precuneus and changes in intermittent pain and PSQI. The precuneus gathers information for somatosensory sensation and is therefore responsible for the recognition of pain sensation (Nagamachi et al., 2006). The precuneus plays a significant role in sleep. Altena et al. reported that reduced gray matter volume was found in the precuneus in patients with insomnia (Altena et al., 2010). One of the reasons for sleep improvement may be related to the changes in the precuneus. The precuneus in a wide range of highly integrated tasks, including visuospatial imagery, episodic memory retrieval, self-processing operations, aspects of consciousness, pain perception, and endogenous pain modulation (Greicius et al., 2009). Groote et al. (2020) suggested the role of the precuneus as a monitoring tool for the effect of SCS. We found an increase in ReHo and DC values in the left precuneus after stSCS; these data may confirm the important role of the precuneus in the alleviation of pain. Depression severity was previously correlated with functional connectivity in the left middle temporal cortex and precuneus (Lassalle-Lagadec et al., 2012). The precuneus is the main hub of the brain and represents a core region of the default-mode network (DMN). The DMN is the resting-state network of the brain and consists of the posterior cingulate cortex, medial frontal, precuneus, posterior parietal cortex, and lateral temporal cortex (Raichle et al., 2001). The DMN is not only responsible for emotional processing, self-introspection, awareness, and the extraction of scenario memory, but also correlative pain suppression (Argaman et al., 2020), functional connectivity hubs, and brain networks (Tomasi and Volkow, 2011). The resting-state DMN is abnormal in patients with chronic painful conditions, thus implicating the impact of such chronic conditions on areas beyond pain perception.

Several brain regions related to emotion were changed after stSCS therapy. The parietal lobe is associated with emotion, sensory, and cognitive function (Lai, 2019). Anxiety is associated with reduced cerebral blood flow in the parietal lobe (Fredrikson et al., 1997). The severity of anxiety is inversely correlated with metabolism in the temporal and parietal regions in patients with mood disorder (Osuch et al., 2000). Improvements in the severity of panic symptoms have also been negatively correlated with the changes in ReHo values in the right superior parietal lobe (Lai and Wu, 2013). Patients with PHN show significantly reduced ReHo values in the right parietal lobe (Cao et al., 2017b). We also observed an increased ReHo value in the superior parietal gyrus after stSCS. It is possible that the superior parietal lobe plays an important role in improving emotions following treatment with stSCS. Moreover, increases in DC and ReHo values in the inferior parietal lobule (IPL), which includes the angular gyrus and the supramarginal gyrus, were observed after stSCS. A significant correlation was identified between DC and ReHo changes in the IPL and changes in the HADS-D and HADS-A. We also identified a significant correlation between DC changes in the left supramarginal gyrus and changes in intermittent pain. It is possible that the IPL may be engaged in attention control, self-awareness, and the regulation of negative affection (Zhuang et al., 2017). The IPL participates in emotional expression and is related to happiness (Schmidt et al., 2020). The angular gyrus is both a node of the DMN and a subregion of the inferior parietal lobule (Gray et al., 2020). The supramarginal gyrus processes external/perceptual information, whereas the angular gyrus processes internal/conceptual information (Rubinstein et al., 2021). The IPL plays a role in episodic memory encoding (Rubinstein et al., 2021). It is possible that stSCS may influence ILP to alleviate pain and emotional function. With regard to emotion, we also found that the ReHo value increased and the DC value decreased in the rolandic operculum after stSCS. There was a significant correlation between DC changes in the rolandic operculum and changes in the HADS, HADS-D, and intermittent pain. One of the reasons why SCS therapy led to an improvement in emotions may be related to the changes in rolandic operculum. Previous studies have reported that the rolandic operculum is associated with psychology and also emotional responses to music (Zhang et al., 2020). Lesions in the right rolandic operculum are known to contribute to worse psychological conditions (high apathy, depression, anxiety, and perceived stress) (Sutoko et al., 2020). The rolandic operculum and IPL may also play the important roles in alleviating the comorbidity of emotion disorder and pain.

The motor function of several brain regions was found to undergo the changes after stSCS therapy. The ReHo value for the superior frontal gyrus was found to increase following stSCS. The human SFG into the anteromedial (SFGam), dorsolateral (SFGdl), and posterior (SFGp) subregions is based on the diffusion tensor tractography (Li et al., 2013). The SFGam is a node of the DMN and is involved in self-referential processing and cognitive control (Northoff et al., 2006). The SFGdl plays a role in the execution of cognitive manipulation (Li et al., 2013). The SFGp is connected with sensorimotor areas (Yu et al., 2011). Regions in the right SFG are implicated in a variety of tasks, including motor movement, working memory, sensorimotor, and cognitive control (Briggs et al., 2020). The concurrent involvement in motor nerves could induce the symptoms of segmental zoster paresis, a condition that is manifested by localized asymmetric myasthenia; the range of this condition generally follows the distribution of myomeres with skin rashes (Meng et al., 2021). SCS has also been shown to alleviate motor deficit (Megía García et al., 2020). The stSCS may exert an effect on the SFGp to alleviate pain and motor function. In this study, the PCG is also involved in motion function in that the DC value of the PCG was increased. The PCG features the whole primary motor area (M1); a very small part of this is located by the most caudal edge of the premotor cortex. The M1 has recently been shown to be a crucial node in the processing of cognitive information related to motor function (e.g., spatial transformations, serial order coding, stimulus-response incompatibility, motor skill learning and memory, and motor imagery) (Georgopoulos, 2000). Changes in the SGF and PCG may represent central mechanisms by which SCS can alleviate motor function although this requires further investigation.

Since several patients withdrew from their consent, the most evident limitation of this study is the relatively small sample size; this may have had an influence on statistical power. Since the patients had a strong desire to accept stSCS therapy to alleviate pain, unfortunately, there is no other treatment control group. Future research should investigate the correlation between motor function and neural activity changes.

## Conclusion

Collectively, this study dynamically and visually investigated the local activity and functional connectivity of brain regions in patients with PHN when treated by stSCS therapy. We demonstrated that after stSCS treatment, patients with PHN exhibit the alterations in ReHo and DC in several brain regions; these regions are related to sensory, motor, memory, sleep, and emotional processes. Moreover, we also observed significant correlations between alterations within these regions and pain intensity, sleep quality, and emotional disorder. This suggests that stSCS therapy may alleviate pain, sleep, and emotional disorder *via* mechanisms in specific brain segments. Our results provide further insights into the mechanisms of stSCS therapy that may activate neurophysiological pathway in the brain.

## Data availability statement

The original contributions presented in the study are included in the article/supplementary material, further inquiries can be directed to the corresponding author/s.

## Ethics statement

The studies involving human participants were reviewed and approved by the Medical Ethics Committee of the First Affiliated Hospital of Zhengzhou University, Zhengzhou, China (Reference: 2020-KY-0299-001). The patients/participants provided their written informed consent to participate in this study. Written informed consent was obtained from the individual(s) and/or minor(s)' legal guardian/next of kin for the publication of any potentially identifiable images or data included in this article.

## Author contributions

XF helped to conceive, design, and examine the manuscript. HR helped to collect, assemble, analyze the data, and write the manuscript. CB helped to collect, assemble, and analyze the data. ZL and LF helped to collect and assemble the data. YW helped to assemble and analyze the data. FX helped to collect and assemble the data and write the manuscript. LM, CK, and TW helped to analyze and interpret the data. YZ helped to analyze the data. QL and WH helped to interpret the data. HB helped to conceive and design. JY helped to design and examine the manuscript. All authors contributed to the article and approved the submitted version.

## Funding

This study was supported by the National Key Research and Development Program of China (grant no. 2020YFC2008400) and the National Natural Science Foundation of China (82001182).

## Conflict of interest

The authors declare that the research was conducted in the absence of any commercial or financial relationships that could be construed as a potential conflict of interest.

## Publisher's note

All claims expressed in this article are solely those of the authors and do not necessarily represent those of their affiliated organizations, or those of the publisher, the editors and the reviewers. Any product that may be evaluated in this article, or claim that may be made by its manufacturer, is not guaranteed or endorsed by the publisher.
